# Anti-sporozoite antibodies as alternative markers for malaria transmission intensity estimation

**DOI:** 10.1186/1475-2875-13-103

**Published:** 2014-03-17

**Authors:** Kwadwo A Kusi, Samuel Bosomprah, Daniel Dodoo, Eric Kyei-Baafour, Emmanuel K Dickson, Daniel Mensah, Evelina Angov, Sheetij Dutta, Martha Sedegah, Kwadwo A Koram

**Affiliations:** 1 Department of Immunology, Noguchi Memorial Institute for Medical Research, College of Health Sciences, University of Ghana, Legon, Ghana; 2 Department of Biostatistics, School of Public Health, College of Health Sciences, University of Ghana, Legon, Ghana; 3 Walter Reed Army Institute of Research, Malaria Vaccine Branch, Silver Spring, MD, USA; 4 Walter Reed Army Institute of Research, US Military Malaria Vaccine Program, Naval Medical Research Center, Silver Spring, MD, USA

**Keywords:** Malaria, Antibody seroconversion rate, Seroreversion rate, Transmission intensity, Sporozoite antigens, ELISA

## Abstract

**Background:**

Reported malaria cases continue to decline globally, and this has been attributed to strategic implementation of multiple malaria control tools. Gains made would however need to be sustained through continuous monitoring to ensure malaria elimination and eradication. Entomological inoculation rate (EIR) is currently the standard tool for transmission monitoring but this is not sensitive enough, especially in areas of very low transmission. Transmission estimation models based on seroconversion rates (λ) of antibodies to *Plasmodium falciparum* blood stage antigens are gaining relevance. Estimates of λ, which is the measure of transmission intensity, correlate with EIR but are limited by long-term persistence of antibodies to blood stage antigens. Seroprevalence of antibodies to sporozoite antigens may be better alternatives since these antigens usually have shorter immune exposure times. The aim of this study was to develop transmission estimation models based on the seroprevalence of antibodies to two *P. falciparum* sporozoite antigens (CSP, CelTOS) and compare with models based on the classical blood stage antigen AMA1.

**Methods:**

Antibody levels in archived plasma from three cross-sectional surveys conducted in 2009 in a low transmission area of Southern Ghana were assessed by indirect ELISA. Seroprevalence of antibodies against CSP, CelTOS and AMA1 were fitted to reversible catalytic models to estimate λ and corresponding seroreversion rates (ρ) for each antibody.

**Results:**

Of the three models developed, the anti-CSP model predicted a 13-fold decrease in λ four years prior to the time of sampling (2009). Anti-AMA1 antibodies formed at a four-fold greater rate compared to that of anti-CelTOS antibodies, and anti-CSP antibodies during the period of decreased λ. In contrast, anti-AMA1 antibodies decayed at a five-fold slower rate relative to that of anti-CSP antibodies while anti-AMA1 and anti-CelTOS antibody decay rates were not significantly different. Anti-CSP antibodies were relatively short-lived as they formed at an 11.6-fold slower rate relative to their decay during the period of decreased λ.

**Conclusions:**

These features of anti-CSP antibodies can be exploited for the development of models for predicting seasonal, short-term changes in transmission intensity in malaria-endemic areas, especially as the elimination phase of malaria control is approached.

## Background

Malaria caused by *Plasmodium* is an infectious disease of public health importance, with an estimated mortality of 655,000 in 2010 [1]. The most severe forms of malaria are usually caused by *Plasmodium falciparum*, and control of the parasite and/or the mosquito vector is vital for disease prevention and elimination. According to the World Health Organization (WHO) [1], the incidence of malaria in most endemic countries, including most parts of Africa, has continued to decrease most likely as a result of the diverse prevention and control measures introduced at the turn of the Century. In order to sustain these efforts and ensure eradication, efficient disease transmission monitoring systems would be required. In endemic areas, the risk of infection is directly related to the intensity of transmission, which has traditionally been estimated by entomological inoculation rates (EIR) or by parasite prevalence estimates using light microscopy [2,3]. These methods however have inherent limitations; EIR estimation is usually laborious, time-consuming, requires very large mosquito samples for robust estimates and has been found to be less accurate in low transmission areas [4,5]. Parasite prevalence estimation is also limited by a reduced detection sensitivity, especially in low transmission areas, as well as by acquired immunity and anti-malarial drug intake which reduce parasite prevalence [6,7]. There is, therefore, a need for less laborious tools with improved reliability and sensitivity for estimating malaria transmission intensity.

Infection with *P. falciparum* elicits immune responses that confer an age and exposure-dependent semi-immunity to infected individuals while being indicative of exposure to parasites. The prevalence of antibodies against such *Plasmodium* antigens as apical membrane antigen 1 (AMA1), the 19 kDa fragment of merozoite surface protein 1 (MSP1_19_) and merozoite surface protein 2 (MSP2) have gained relevance as transmission monitoring tools [8-10]. In areas of stable medium to high transmission, antibody prevalence estimates correlate well with standard EIR estimates, but have an advantage over EIR estimation since antibody decay is slower than parasite clearance rates. The persistence of antibodies long after transmission has ceased however represents a weakness in this approach [11-13], especially if models are to be used for the prediction of seasonal or short-term changes in transmission. A careful selection of antigens and adjustment for antibody persistence in estimation models are therefore necessary under these conditions.

Unlike merozoites, the sporozoite stages of *P. falciparum* are exposed to the immune system for only short periods after inoculation, and anti-sporozoite antibodies would most commonly be detected in individuals with frequent or recent exposure. Cell-traversal protein for ookinetes and sporozoites (CelTOS) and circumsporozoite protein (CSP) are important sporozoite antigens that are relatively more conserved compared to merozoite surface antigens [14-16] and may be ideal candidates for estimating malaria transmission intensity. Estimates based on these antigens, which have short immune exposure times, might therefore better assess differences in exposure to parasites while eliminating the need for considering long-term antibody persistence and antigen polymorphism. This approach will also measure direct exposure to sporozoites similar to the currently available EIR gold standard, as opposed to models based on blood stage antigens, which may not reflect direct sporozoite exposure. An accurate estimation of malaria transmission intensity is crucial as it will permit an assessment of the effectiveness of interventions and aid in planning in the framework of the limited resources that are available to most disease-endemic countries so that current gains are not eroded.

The aim of this study was therefore to develop and compare transmission estimation models based on the seroprevalence of antibodies against the parasite antigens CSP, CelTOS and AMA1 using archived plasma samples from individuals living in a low malaria transmission area. The study found that of the three antigens, CSP represents a potentially useful sero-epidemiological marker for predicting short-term changes in transmission intensity as anti-CSP antibodies were relatively non-persistent in the study population. Estimation models based on anti-CSP antibody seroprevalence predicted a 13-fold decrease in transmission intensity in the study area four years prior to the sampling time, around 2005. Models based on CelTOS and AMA1 antibodies however did not predict changes in malaria transmission intensity.

## Methods

### Ethics statement

The current study used archived plasma samples from a previous cohort study that was conducted in 2009. Ethical approval for the parent study was granted by the Institutional Review Board (IRB) of the Noguchi Memorial Institute for Medical Research (NMIMR). Study participants or their parents/legal guardians gave written informed consent, which included an agreement for storage and use of their samples for future malaria studies, before enrolment into the study. For the present study, analysis of archived plasma samples was done blinded.

### Study site and sampling

The current study utilized archived plasma samples from an earlier study carried out in Asutsuare in the Dangme West district of the Greater Accra Region of Ghana. Asutsuare is a rural community about 120 km northeast of the Ghana capital, Accra, and the majority of inhabitants are peasant farmers. The community has a network of canals for irrigation purposes and like most other communities in the district Asutsuare has two peak rainfall seasons. The major rainy season occurs from March to August and the minor rainy season occurs in November to December, followed by a period of very dry conditions. The area has served as a site for a number of malaria intervention-related studies since the early 1990s. A total of 560 participants aged from one to 30 years were recruited for the original study, and three cross-sectional community-based surveys were conducted in February, May and August of 2009 to collect blood samples for laboratory analysis. For the current study, samples from 294 participants were randomly selected from the original 560. Archived plasma samples from the same 294 participants for all three cross-sectional survey time points were assayed.

### Antigens and plasma antibody determination

Antigens used in this study were all from the 3D7 strain of *P. falciparum* and were expressed and purified under GMP conditions. The near full-length CSP protein containing 19 of the 38 NANP repeats was expressed in an *Escherichia coli* system [17]. The CelTOS protein is comprised of 174 amino acids including an N-terminal six-histidine tag within a 16-amino acid linker [18]. The AMA1 protein is comprised of amino acids 83 to 531 of the AMA1 ectodomain and was also expressed in *E. coli*[19,20].

Plasma levels of antibodies to the three parasite antigens were determined by an indirect ELISA protocol. Briefly, 96-well ELISA plates (Maxisorp, NUNC, Denmark) were coated with 100 μl/well of 1 μg/ml of the respective antigens in PBS, pH 7.2 and the plates incubated overnight at 4°C. After overnight incubation, plates were blocked with 200 μl/well of 3% non-fat milk in PBS, 0.05% Tween 20 for one hour at room temperature. Plates were subsequently incubated with 100 μl/well of duplicate test plasma samples diluted 1:1,000 in 1% non-fat milk in PBS. A pool of semi-immune sera was used as a standard calibrator sample (diluted 50 times for CSP, CelTOS and 5,000 times for AMA1) and titrated on each plate as an internal control. Plates were incubated for two hours at room temperature, and subsequently incubated with 100 μl/well of a 1:50,000 dilution of goat anti-human IgG conjugated to horseradish peroxidase (Invitrogen, CA, USA) for another hour at room temperature. After enzyme conjugate incubation, plates were developed with TMB (KEM-EN-TEC, Taastrup, Denmark) as substrate for 15 min at room temperature and the colour reaction was stopped by the addition of 50 μl/well of 0.2 M H_2_SO_4_. Optical densities (ODs) were subsequently read at 450 nm on a 96-well ELISA plate reader (BioTek, VT, USA). In between all incubation steps, plates were washed five times with PBS, pH 7.4, 0.05% Tween 20 using an automated plate washer.

### Statistical analysis

Differences in the levels of antibodies against the three antigens were assessed by comparing ODs. Data for each antigen were split into three age categories based on the ages of study participants: one to five years, six to 15 years and 16 – 30 years. For each antigen, the Kruskal-Wallis test on ranks was used for comparison of ODs at the different sampling time points, and this was followed by pair-wise comparisons using Bonferroni post-hoc tests where necessary.

For each coating antigen, a mixture model was used to define a cut-off value above which samples were deemed antibody-positive as previously described [21]. Briefly, the distribution of normalized OD values was fitted as the sum of two Gaussian distributions; a narrow distribution of seronegatives and a broader distribution of seropositives, using maximum likelihood methods. The mean OD of the Gaussian corresponding to the seronegative population, plus three standard deviations, was used as the cut-off for seropositivity. Seroprevalence was calculated as the proportion of samples with OD above this cut-off. The seroconversion rate (SCR or **λ**), which is the rate at which individuals change from being seronegative to seropositive, represents exposure to malaria parasites over time and is analogous to the force of infection [9]. For each cross-section, **λ** was estimated using a catalytic conversion model, assuming a binomial error distribution [22], to fit data on seroprevalence by subject age. The model calculates **λ** and the seroreversion rate (**ρ**; ie., the rate at which seropositive individuals revert back to seronegativity). Seroprevalence curves were plotted using observed seropositivity data and the median age of ten age groups, each of which is a centile of the observed data. The fitted values and 95% confidence limits were also plotted and the resulting **λ** is presented for each cross-sectional time point. Evidence for temporal changes in **λ** was explored by fitting models in which **λ** is allowed to change at a single time-point. Likelihood ratio test was used to assess the significance of the change in **λ** against models with no change. Profile likelihood plots were used to determine the most likely time that a change in **λ**, and hence transmission intensity, occurred. Analysis of data and graphics were performed using Stata (Statacorps, TX, USA) and R (R development Core Team).

## Results

The study utilized plasma samples from three cross-sectional blood surveys of individuals living in Asutsuare, an area of low and seasonal malaria transmission in southern Ghana. A total of 882 archived plasma samples, three each from 294 participants aged between one and 30 years were analysed. Of the 294 participants, 114 were one to five years old, 144 were six to 15 years old and 36 were 16 – 30 years old. Parasite prevalence in the study population, estimated by light microscopy, was 2.4% for the first survey in February 2009. This increased slightly to 2.7% in May 2009 and returned to 2.4% for the third survey in August 2009. Parasitaemia was limited to the two younger age groups (on average, three of the seven participants with parasitaemia were one to five years old and the remaining four parasitaemic participants were six to 15 years old) at all three time points.

**Figure 1:**
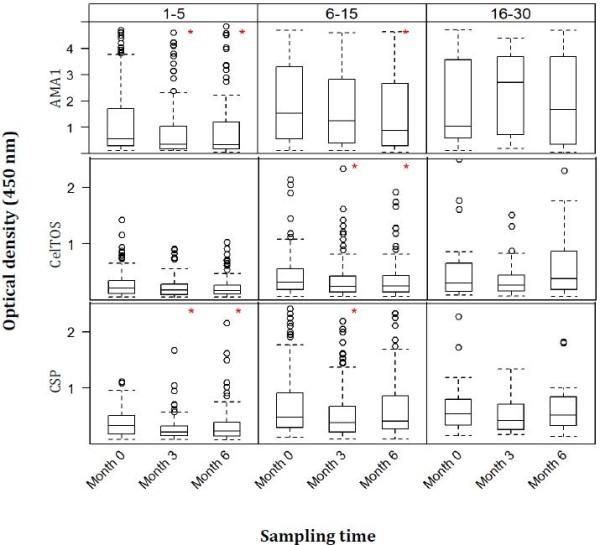
**Comparison of OD**_**450 nm **_**by cross-sectional sampling times for all three antigens.** Antigen-specific optical density (OD) values at the three cross-sectional time points (months 0, 3 and 6) were compared within each age group using Kruskal-Wallis Analysis of Variance by Ranks tests, followed by Bonferroni post-hoc tests where necessary. Month 0 is the dry season while months 3 and 6 represent the major and minor rainy seasons, respectively. Boxes represent the median, lower quartile (25^th^ percentile) and upper quartile (75^th^ percentile) of each data subset, and whiskers are 1.5 times the interquartile range. Red asterisks (*) indicate a statistically significant difference (p value <0.05) in median OD between month 0 and any of the two other time points (month 3 or month 6) following pair-wise comparisons. No significant differences were observed between month 3 and month 6 OD data for all three antigens following the post-hoc tests.

For each of the three capture antigens, measured ODs were generally lower in the one to five years old group compared to the two other age groups (six to 15 years and 16–30 years) at any cross-sectional time point as is expected (Figure 1). There were no significant differences between ODs of parasite-positive and parasite-negative volunteers for any of the antigens and age groups.

OD data at a 1:1,000 dilution of plasma samples was compared amongst the three antigens for each cross-sectional time point. Median ODs were always significantly different amongst the three antigens (p <0.05, Bonferroni post-hoc test on ranks), with the blood stage antigen AMA1 having the highest median OD at all three time points.

### Comparison of antigen-specific OD data at the different sampling time points

#### Anti-AMA1 responses amongst the different age groups

Median OD against AMA1 was significantly higher at month 0 (February 2009) when compared pair-wise with those at month 3 (May 2009) and month 6 (August 2009) for the one to five years old group (p < 0.05 in both cases, Bonferroni post-hoc tests, Figure 1). Median OD was also significantly higher at month 0 compared to month 6 in the six to 15 years old group (p = 0.024, Bonferroni post-hoc test). Median ODs at the three time points were however not significantly different in the 16–30 years old group (p = 0.67, Kruskal-Wallis test, Figure 1).

#### Anti-CelTOS responses amongst the different age groups

There was no statistically significant difference in median OD against CelTOS at the three cross-sectional time points in both the one to five years old group (p = 0.11, Kruskal-Wallis test, Figure 1) and the 16–30 years old group (p = 0.30; Kruskal-Wallis tests). Median OD was however significantly higher at month 0 when compared separately with month 3 (p = 0.035) and month 6 (p = 0.028) in the six to 15 years old group.

#### Anti-CSP responses amongst the different age groups

As was the case with AMA1, median OD against CSP was significantly higher at month 0 when compared pair-wise with those at months 3 and 6 for the one to five years old group (p < 0.05 in both cases, Figure 1). For the six to 15 years old group however, the median OD at month 0 was only significantly higher compared to that at month 3 (p = 0.003). Median ODs against CSP at the three time points were also comparable in the 16–30 years old group (p = 0.30, Kruskal-Wallis test).

#### Antigen-specific seropositivity and rainfall patterns

**Figure 2:**
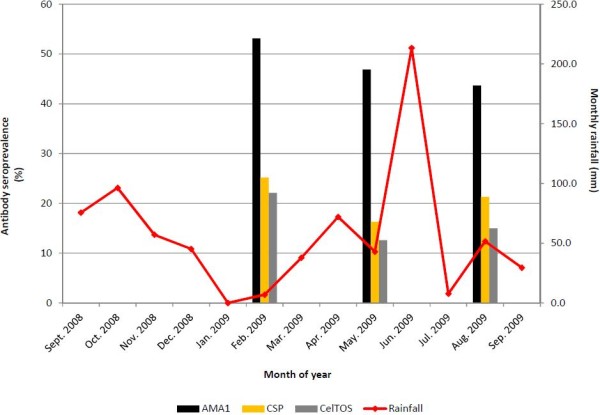
**Proportion of seropositive individuals and monthly rainfall over study period.** For each antigen, seropositivity was defined using a mixture model, which involves maximum likelihood estimation of seronegative individuals from a Gaussian distribution of OD data for each antigen. No statistically significant differences were found amongst the proportions seropositive at the three time points for any of the three antigens.

The proportions of individuals who were seropositive for each antigen at the three cross-sectional time points are presented along with monthly rainfall data for the study area, obtained from the Ghana Meteorological Service, in Figure 2. Seropositivity against AMA1 was 53.1% at month 0 and decreased progressively at month 3 (46.9%) and month 6 (43.7%), whilst seropositivity against CSP decreased from 25.2% at month 0 to 16.3% at month 3, but increased slightly to 21.3% at month 6. As was the case for CSP, seropositivity against CelTOS decreased from 22.1% at month 0 to 12.6% by month 3, before reaching 15.0% by month 6 (Figure 2). These changes in seropositivity over time were however not significantly different for any of the antigens.

### Fitting of seroprevalence curves by the reversible catalytic model

**Figure 3:**
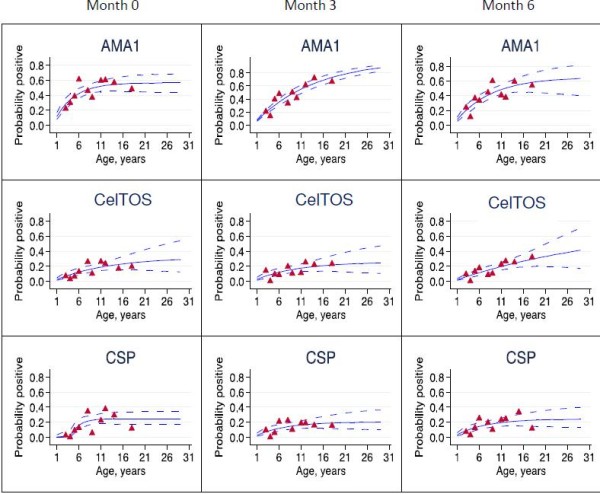
**Seroprevalence curves for all *Plasmodium falciparum *antigens at each cross-section.** Seroprevalence curves represent the rate at which individuals become seropositive to specific antigens. In each graph, points represent age seroprevalence (divided into deciles), unbroken lines represent maximum likelihood curves and broken lines represent 95% confidence intervals.

**Figure 4:**
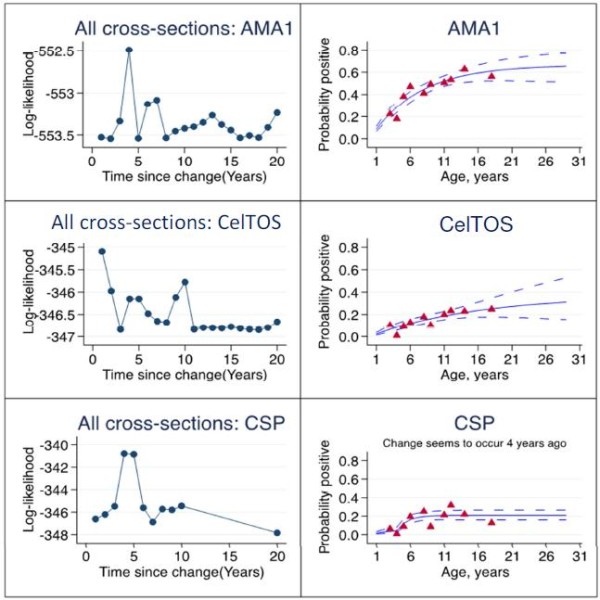
**Log-likelihood profiles for reversible catalytic models of probability seropositive to AMA1, CelTOS and CSP.** Models incorporate data from all three cross-sectional time points and allow for abrupt changes in transmission intensity at some time point.

Seroprevalence data for each antigen were fitted using reversible catalytic models; seroprevalence curves for each cross-sectional time point are presented in Figure 3 and seroprevalence curves incorporating data from all three time points for each antigen, with a correction for clustering, are presented in Figure 4.

While seroprevalence curves for antibodies to AMA1 and CelTOS at all sampling time points were mostly monophasic, the CSP-based model for month 0 had a biphasic seroprevalence curve, suggesting a change in **λ** in the study area at some previous time point. Maximum log likelihood estimates based on month 0 anti-CSP seroprevalence data indicated a ten-fold decrease in **λ** occurred approximately four years prior to the time of sampling (before change, λ_1_ = 0.226; after change, λ_2_ **=** 0.022, p <0.01; Table 1, Figure 3). The overall CSP model, incorporating data from all three time points also predicted a 13-fold decrease in **λ** around the same period (before change, λ_1_ = 0.319; after change, λ_2_ **=** 0.024, p < 0.01; Table 1, Figure 4).

**Table 1:** Seroconversion and seroreversion rates for models based on AMA1, CelTOS and CSP

**Antigen**	**Month of survey**	**Number of subjects (N = 882)**	**Seroconversion rate (95% CI)**	**Seroreversion rate (95% CI)**
AMA1	February	294	0.144 (0.089, 0.235)	0.131 (0.057, 0.302)
	May	294	0.074 (0.051, 0.108)	0.005 (0.000, 51.872)
	August	294	0.078 (0.051, 0.118)	0.034 (0.005, 0.223)
^**§**^Total (AMA1)			0.095 (0.070, 0.129)	0.055 (0.022, 0.135)
CelTOS	February	294	0.026 (0.013, 0.054)	0.057 (0.005, 0.609)
	May	294	0.027 (0.012, 0.059)	0.079 (0.010, 0.617)
	August	294	0.020 (0.015, 0.027)	0.0000002 (0.00, 4718.9)
^**§**^Total (CelTOS)			0.023 (0.013, 0.041)	0.036 (0.002, 0.552)
CSP	February	294	λ_1_ = 0.226 (0.073, 1.700)	0.171 (0.093, 0.311)
		*LRT: P < 0.01	λ_2_ = 0.022 (0.012, 0.040)	
	May	294	0.033 (0.014, 0.077)	0.132 (0.029, 0.595)
	August	294	0.028 (0.014, 0.055)	0.054 (0.006, 0.512)
^**§**^Total (CSP)		*LRT: P < 0.01	λ_1_ = 0.319 (0.084, 1.219)	0.278 (0.166, 0.464)
			λ_2_ = 0.024 (0.013, 0.044)	

Seroconversion and seroreversion rates are estimated from seroprevalence curves for each antigen (Figures 3 and 4).

^**§**^Reversible catalytic model with standard error adjusted for clusters due to repeated cross-sectional sampling.

*LRT represents likelihood ratio test of no change in seroconversion rate.

### Comparison of seroconversion and seroreversion rates for each antigen

For models that include data from all three time points, the decay rate of anti-CSP antibodies (ρ = 0.278, 95% CI 0.166 - 0.464) was not significantly different from the seroconversion rate of anti-CSP antibodies in individuals born before the time of predicted change in **λ** (λ_1_ = 0.319, 95% CI 0.084-1.219). The decay rate of anti-CSP antibodies was however 11.6-fold greater than the seroconversion rate of the same antibodies in individuals born after the time of predicted change in **λ** in the study population (λ_2_ = 0.024, 95% CI 0.012-0.044). The rates of induction and decay of anti-CelTOS and anti-AMA1 antibodies were not significantly different (Table 1).

### Comparison of seroconversion rates (λ) amongst the three antigens

Comparison of models including data from all three time points showed that anti-AMA1 antibody seroconversion (λ = 0.095, 95% CI 0.070-0.129) was comparable to that of anti-CSP antibodies before the time of predicted change in **λ** (λ_1_ = 0.319, 95% CI 0.084-1.219) but was four-fold higher than that for anti-CSP antibodies after the time of predicted change in **λ** (λ_2_ = 0.024, 95% CI 0.012-0.044). Anti-AMA1 antibody seroconversion was also at a four-fold greater rate than that of anti-CelTOS antibodies (λ = 0.023, 95% CI 0.013-0.041). Anti-CSP antibody seroconversion rate before the change in **λ** was up to 14-fold higher than that of anti-CelTOS antibodies, but there was no significant difference between anti-CSP seroconversion rate after the time of predicted change in **λ** and that of anti-CelTOS antibodies (Table 1).

### Comparison of seroreversion rates (ρ) amongst the three antigens

Comparison of corresponding antibody decay rates for data from all sampling time points showed that anti-CSP antibody seroreversion (ρ = 0.278, 95% CI 0.166-0.464) was at a five-fold greater rate than that for anti-AMA1 antibodies (ρ = 0.055, 95% CI 0.022-0.135). Anti-CSP antibodies also decayed at an eight–fold greater rate than that of anti-CelTOS antibodies (ρ = 0.036, 95% CI 0.002-0.552). There was, however, no significant difference in the decay rates of anti-CelTOS and anti-AMA1 antibodies (Table 1).

## Discussion

As an increasing number of malaria-endemic countries approach the elimination phase of malaria control, more sensitive tools will be required to detect the remaining hotspots of disease transmission in order to implement targeted control. Such tools will also help to detect resurgences in transmission intensity in areas where control measures become lax. A number of studies have described sero-epidemiological transmission estimation models based on the prevalence of antibodies to blood stage antigens, such as MSP1, MSP2 and AMA1 [8-10,23,24]. Although this might be a potentially more sensitive tool in areas of very low transmission, antibody responses, especially to blood stage antigens, have been shown to persist for years to decades after transmission has ceased [12,13,25,26]. Thus antibodies to blood stage antigens may still be present in easily detectable quantities in populations where there is no ongoing malaria transmission. Antigens from the sporozoite stages of *Plasmodium* usually have shorter immune exposure time and are less immunogenic, and antibody levels to these antigens are expected to be relatively non-persistent compared to those against blood stage antigens. The aim of this study was therefore to develop transmission intensity estimation models based on the seroprevalence of antibodies against sporozoite antigens (CelTOS and CSP) and compare to models based on the classical blood stage antigen AMA1.

The current data confirm the general observation that plasma levels of antibodies against sporozoite antigens (CelTOS and CSP) are comparatively lower than levels against blood stage antigens in populations with natural exposure to malaria [27]. For any of the three antigens, comparisons of OD data by sampling time point show that antibody levels were generally higher at month 0 compared independently to months 3 and 6 in the two younger age groups (one to five and six to 15 years) whilst antibody levels did not change significantly with time in the 16–30 years old group (Figure 1). These observations might be related to the limited exposure of study subjects to parasites as less than 3% of the study population had blood film parasites. Thus, antibody boosting was minimal and variations in antibody levels will mostly be observed in the younger age groups with limited pre-existing malarial immunity, in contrast to older individuals who are expected to have a semi-immune status following repeated exposure to parasites [28,29]. Indeed, all individuals who had blood film parasites were in the two younger age groups. The possible presence of submicroscopic infection in a good proportion of study participants can however not be ruled out, but long term submicroscopic infection in the absence of exposure to new infectious bites may only have a role in the induction/maintenance of antibodies against blood stage antigens [30] but not sporozoite antigens.

Seropositivity against all three antigens was not significantly different across the three sampling time points (Figure 2). There was therefore no association between antibody seropositivity and the levels of rainfall recorded at each time point, and this may once again be explained by the low infection rates measured in this study.

Maximum likelihood estimates from reversible catalytic models showed that seroconversion rates for antibodies against the two sporozoite antigens (CelTOS, and after 2005 CSP) were four-fold lower than that of antibodies against the typical blood stage antigen AMA1 (Table 1). Also, anti-CSP antibodies waned at a significantly faster rate (five-fold) than the rate at which anti-AMA1 antibodies waned, but there was no significant difference between anti-CelTOS and anti-AMA1 decay rates (Table 1). In addition, decay rate for anti-CSP antibodies was about 11.6-fold greater than the rate at which the same antibodies formed after the time of predicted change in **λ**. These observations collectively suggest that anti-sporozoite antibodies, especially those against CSP, are formed at a relatively slow rate and decay at a faster rate compared to anti-AMA1 antibodies in individuals from low transmission areas. Anti-CSP antibodies are therefore less likely to persist for very long periods as is the case for anti-AMA1 antibodies in this study and indeed for antibodies to other blood stage antigens [12,24-26,31]. Thus, the relatively short-lived anti-sporozoite antibodies will mostly be detected in individuals with recent exposure to sporozoites, and this might make anti-CSP antibodies very good markers of exposure to recent infectious bites. Indeed, of the three models developed in the current study, the CSP model for the first cross-sectional survey estimated a ten-fold decrease in **λ** as a proxy for transmission intensity (Table 1, Figure 3), while the CSP model incorporating data from all three sampling time points demonstrated a 13-fold decrease in **λ** (Table 1, Figure 4), occurring four years prior to the time of sampling.

The association of anti-CSP antibody prevalence with the extent of malaria transmission in this study corroborates data from other studies in parts of Africa and Asia [13,32,33]. Anti-CSP antibody prevalence has also been shown to parallel seasonal transmission patterns determined by parasitologic and entomologic measurements in Southeast Asia [34]. Empirical data from the study area in turn corroborates findings in the current study. Data gathered in the 1990s show that parasite prevalence estimated by light microscopy amongst members of the larger community could be as high as 51% during the rainy season and 20% in the dry season [35], whilst more recent data collected after year 2010 generally show blood film parasite prevalence of less than 6% (Quartey, unpublished data). Indeed, the current study with plasma samples collected from February to August 2009 showed parasite prevalence of 2.4-2.7% over the study period. This decrease in parasite infection rates in the study area could be attributed to the impact of continuous malaria intervention efforts that have been ongoing in the district since the early 1990s. The district has been involved in the piloting and testing of several malaria intervention programmes, including mass anti-malarial drug administration, distribution of insecticide-treated bed nets to households, improvement in disease monitoring and malaria case management at hospitals/clinics and within communities [36-41]. The district was also one of the first in Ghana to implement the Global Fund-supported Roll Back Malaria programme, which began in 1999. The study area, in addition, is one of the rural communities in the Greater Accra region with the highest number of Community-based Health Planning Services (CHPS) compounds, a health improvement concept that is run by the Ministry of Health in Ghana to provide immediate healthcare services and education to people in rural and remote communities [42]. These various factors have most likely made current malaria control tools more easily accessible to residents, with the result being a drastic decrease in transmission intensity in this area.

Transmission estimation models based on month 3 and month 6 anti-CSP seroprevalence data were unable to predict changes in **λ**, in contrast to the CSP model based on the month 0 survey. Although the reason for this is not clear from the data, one important difference between the three surveys is that the first was conducted in the dry season whilst the other two surveys were conducted during the rainy season of 2009. It is possible that anti-CSP seroprevalence dynamics under these two environmental conditions could be different, and this may influence the ability of the model based on the month 0 (February 2009) survey to predict changes in **λ** while the other two models (May and August 2009) do not. If this observation is confirmed, it would have implications for the timing of surveys for antibody seroprevalence model development.

From the modelling data, anti-CSP antibody seroconversion rates were different under low and high transmission settings, while that of anti-CelTOS antibodies was not dependent on transmission intensity (Table 1), hence the model based on anti-CelTOS antibodies did not predict changes in **λ**. Although the reason for this observation is unclear, it could be attributed to the relatively lower level of anti-CelTOS antibody seroconversion compared to that of anti-CSP antibodies during predicted periods of high transmission. This may be related to differences in the immunogenicity and/or relative abundance of the two antigens (CSP, CelTOS) on the sporozoite surface [43].

## Conclusions

This study has demonstrated the potential of sporozoite antigens, especially CSP, as important markers for assessing changes in malaria transmission intensity. The transmission estimation model based on CSP predicted a 13-fold decrease in transmission intensity in the study area, occurring approximately four years prior to the time of sampling. The study data showed that anti-AMA1 antibodies were formed at a rate that is at least four-fold greater compared to that of antibodies against the two sporozoite antigens. For anti-CSP antibodies, this relation holds only during the predicted period of decreased transmission as anti-CSP antibody seroconversion rate was not significantly different from that of anti-AMA1 antibodies during periods of high transmission. In contrast, anti-CSP antibodies decayed at a five-fold greater rate relative to that of anti-AMA1 antibodies. Anti-CSP antibodies were shown to decay at a rate that was 11.6-fold greater relative to anti-CSP antibody induction under low transmission settings. Thus under low transmission settings, anti-CSP antibodies formed at a slower rate and decayed at a faster rate compared to anti-AMA1 antibodies, and this is direct evidence of the short-lived nature of anti-CSP antibodies compared to antibodies against typical blood stage antigens. This short-lived nature of anti-CSP antibodies will thus permit their detection in high levels only in individuals with recent exposure to infectious bites. The data therefore highlight the potential of antibodies against CSP as a useful marker for predicting seasonal, short-term changes in malaria transmission intensity.

## Competing interests

The authors declare that they have no competing interests.

## Authors’ contributions

KAK, DD, MS, and KAK conceived and designed the study; KAK, EKB, EKD, and DM performed immunological assays; EA and SD purified and provided recombinant antigens; KAK and SB performed statistical/data analysis; KAK, SB, DD, MS, EA, SD, and KAK wrote the paper. All authors have read and approved the final manuscript.
